# Study on the Inhibitory Effects of Ephedra Aconite* Asarum* Decoction on LPS-Induced Dendritic Cells

**DOI:** 10.1155/2017/3272649

**Published:** 2017-11-26

**Authors:** Wenting Ji, Jinghong Hu, Xue Yu, Lanlan Zhang, Min Liu, Qingguo Wang

**Affiliations:** Beijing University of Chinese Medicine, Beijing 100029, China

## Abstract

Dendritic cells (DCs) can secrete cytokines stimulated by lipopolysaccharide (LPS), which leads to not just acute inflammatory responses but also Th1 polarization. Furtherly, chronic inflammation or autoimmune diseases could be triggered. As a classic Traditional Chinese Medicine formula, Ephedra Aconite* Asarum* Decoction with the main ingredients of ephedrine and hypaconitine can show effect on anti-inflammation and immunoregulation. But it remains unclear whether Ephedra Aconite* Asarum* Decoction controls DCs. In this study, we investigated the effects of Ephedra Aconite* Asarum* Decoction on LPS-induced bone marrow-derived DCs (BMDCs)* in vitro*. We found that Ephedra Aconite* Asarum* Decoction lowered surface costimulators on DCs and reduced the expression of Th1 type cytokines. Yet it is slightly beneficial for shifting to Th2. Our work reveals that the Ephedra Aconite* Asarum* Decoction can regulate Th1 inflammation through intervening DCs.

## 1. Introduction

LPS is a bacterial wall component of the gram-negative pathogens and a major component of endotoxin. It can cause tissue infiltration of inflammatory cells, activation of macrophages, endothelial cells and DCs, and release of proinflammatory cytokines such as IL-12, IL-1, and IFN-*γ*, leading to acute inflammation [1–3]. Meanwhile, cytokines including IFN-*γ* and IL-12 can also promote differentiation of T cells to the Th1 direction [4, 5], while overproduction of Th1 cytokines (such as IL-12 and IFN-*γ*) will lead to chronic inflammation, organ-specific autoimmune responses, and contact dermatitis [6].

DCs are antigen presenting cells (APC) with the strongest antigen presentation ability, which activate T cells and regulate T cell differentiation [7]. DCs are at the center of initiating, regulating, and maintaining immune responses [8]. The function of DCs is closely related to its maturation status. Cell surface expression of CD83, CD80, MHCII, and other costimulators significantly increases upon DCs maturation [9]. In general, the increase of CD83 can be used as a reliable indicator of DCs maturation [10]. Mature DCs play a powerful antigen presenting role to activate T cells and to secrete a variety of cytokines that affect T helper (Th) cell differentiation. Thus, DCs are the key to the pathogenesis of many autoimmune diseases and may also be a main therapeutic target for these diseases [11].

Signal transducers and activators of transcription (STAT) are a family of cytoplasmic proteins with signal transduction and transcriptional activation functions. STAT1, STAT2, STAT3, STAT4, STAT5, and STAT6 are the main members of the STAT family [12, 13]. Except the limited distribution pattern of STAT4, the rest of the STAT proteins are widely distributed among different cell types and tissues.

Among them, STAT6 is closely related to Th2 differentiation of the CD4^+^T cells [14, 15]. STAT6 can activate GATA-3 through the Il-4-STAT6 pathway and promote T cell differentiation towards Th2 cells. T-bet and GATA-3 are the transcription factors of Th1 and Th2 differentiation, respectively, and they guide the specific differentiation of T cells. In addition, GATA-3 is an upstream molecule to STAT6, so T-bet and GATA-3 together promote preferential differentiation of T cell to Th2 cells.

Ephedra Aconite* Asarum* Decoction was made of three herbs including Ephedra, Aconite, and* Asarum*. It has the advantages of precise ingredient compatibility and comprehensive efficacy for the targeted treatment of Shaoyin exogenous syndromes based on its effects in supporting, dispelling, and invigorating Yang as described in the treatise on Febrile Diseases [16–20]. Ohhashi from Japan claimed that this formula is a “special cure” for AR (Allergic Rhinitis) [21], and there are many case reports and clinical observations from China that also show similar efficacies [22, 23]. Modern pharmacological studies found that Ephedra Aconite* Asarum* Decoction had broad indications, as it is commonly used in the clinic for the treatment of AR [24], cold [25], arrhythmia [26], asthma [27], and other diseases and showed great efficacy. The major active ingredients of Ephedra Aconite* Asarum* Decoction include ephedrine, aconite total alkaloids, and* Asarum* volatile oil [28, 29] among others. Studies have shown that ephedrine, aconite total alkaloids, and* Asarum* volatile oil had anti-inflammatory and immunomodulatory effects [30, 31]. In this study, we investigated the impact of Ephedra Aconite* Asarum* Decoction on LPS-induced DCs to explore the effects of this composite formula in LPS-induced inflammatory model.

## 2. Methods

### 2.1. Animals and Materials

Twenty SPF male C57BL/6 mice of 6 weeks old (20 ± 5 g) were purchased from SPF (Beijing) Biotechnology Co., Ltd. All mice were maintained under specific pathogen-free conditions.

### 2.2. Preparation of Ephedra Aconite* Asarum* Decoction Water Extract

Ephedra Aconite* Asarum* Decoction was prepared as follows (usual dose for adults): Ephedra 6 g, Aconite 9 g, and* Asarum* 3 g (this dose is the commonly used clinical dose), boil with the right drug to water ratio in ultrapure water for 1 hour until final drug concentration reaches 1 g/ml. Centrifuge at high speed for 2 times, 12,000 rpb/min, 10 min each, collect supernatant, filter with 0.22 *μ*m filter for sterilization, aliquot, and store at −20°C for future use.

### 2.3. Isolation and Culture of DCs from Mouse Bone Marrow

Methods for DCs isolation and culture from C57BL/6J mice which were raised in the Animal Laboratory of Environmental Barriers System were described in previous literatures [32, 33]. Specifically, isolate femur and tibia of the mouse under sterile condition, aspirate 1 ml RPMI-1640 medium with syringe and flash the bone marrow into a dry sterile culture dish, collect bone marrow cell suspension, lyse red blood cells with ACK buffer, and resuspend cells in RPMI-1640 complete medium containing GM-CSF (10 ng/ml, R&D), IL-4 (10 ng/ml, R&D), 10% FBS, 1% PS, plate cells in 6-well plates at a density of 2 × 10^6^ cells/ml, culture at 37°C in 5% CO_2_, change culture medium every half day. At day 6, cells were harvested, and DCs were stained with APC-conjugated anti-CD11c (HL3) antibody before analysis with a FACS Canto II (BD Biosciences) to identify the purity and maturation of DCs. These cells were used as immature mouse bone-marrow-derived DCs (IMDCs).

### 2.4. CCK-8 Cytotoxicity Test to Screen the Appropriate Concentration of Ephedra Aconite* Asarum* Decoction for Intervention

IMDCs were cultured and cell density was adjusted to 5 × 10^5^ cells/ml, pipette into even single cell suspension, plate into 96-well plate at 100 *μ*l per well, add 10x series dilution of 1 g/ml Ephedra Aconite* Asarum* Decoction water extracts into 96-well plate, make hexplicate of each concentration of water extract, place the cell culture plate in 37°C, 5% CO_2_, saturated humidity incubator for 24 hours, then add 10 *μ*l Cell Counting Kit-8 (CCK-8) reagent to each well, read on microplate reader every half hour, and record the value; next, adjust concentration of the water extract and repeat the experiment until an appropriate concentration of Ephedra Aconite* Asarum* Decoction was chosen for subsequent experiments.

### 2.5. Effects of Ephedra Aconite* Asarum* Decoction on the Expression of CD83, MHCII, and CD80 on LPS-Induced DCs

IMDCs were plated into 24-well plate at 1 × 10^6^ cells/ml, 500 *μ*l per well, set 3 experimental groups including blank control group (group C), LPS model group (group L) as model group, and Ephedra Aconite* Asarum* Decoction group (group M). Groups L and M were stimulated with LPS (1 *μ*g/ml, Sigma), and group M was treated with additional Ephedra Aconite* Asarum* Decoction (2 mg/ml). Groups C and L were treated with equal volume of 1640 as control, harvest cells after 24 h. Cells were stained with FITC-conjugated anti-CD80 antibody (16-10A1, Biolegend), APC-conjugated anti-CD11c antibody (HL3, BD), PE-conjugated anti-CD83 antibody (MICHEL-19, BD), and PE-Cy5.5-conjugated anti-MHCII antibody (M5/114, BD) before analyzing with a FACS Canto II (BD Biosciences).

### 2.6. Effects of Ephedra Aconite* Asarum* Decoction on the Expression of STAT6 and pSTAT6 in LPS-Induced DCs

IMDCs in culture were plated into 6-well plate at 2 × 10^7^ cells/ml, 2 ml per well, set 3 experimental groups including group C, group L, and group M. Groups L and M were induced with LPS (1 *μ*g/ml, Sigma), and group M was also treated with Ephedra Aconite* Asarum* Decoction (2 mg/ml), harvest cells after 24 hours. Clearly, group L was the model control group. Total protein was extracted from each group and expressions of STAT6 (Proteintech) and pSTAT6 (phospho Y641, Abcam) were detected in each group.

### 2.7. Effects of Ephedra Aconite* Asarum* Decoction on the mRNA Expression of GATA-3 and T-Bet in LPS-Induced DCs

IMDCs in culture were plated into 24-well plate at 1 × 10^6^ cells/ml, 500 *μ*l per well, set 3 experimental groups including group C, group L, and group M. Groups L and M were stimulated with LPS (1 *μ*g/ml, Sigma), and group M was also treated with Ephedra Aconite* Asarum* Decoction (2 mg/ml), harvest cells after 24 hours. Also, group L is the model control group. Total ribonucleic acid (RNA) was isolated using TRIzol Reagent (Life Technologies, Paisley, UK) according to the manufacturer's protocol. The cDNA templates were synthesized with a MJ Research PTC-100. Quantitative real-time PCR analyses were performed with CFX96 PCR System (Bio-Rad). Expression levels of target genes were normalized to endogenous *β*-actin transcription levels, and relative quantification of samples was compared with the expression level of group C. We used the following primers, designed by Sangon Biotech (Shanghai) Co., Ltd.: GATA-3: forward primer: 5′-GAAGGCATCCAGACCCGAAAC-3′ and reverse primer: 5′-ACCCATGGCGGTGACCATGC-3′; T-bet: forward primer: 5′-GGTGTCTGGGAAGCTGAGAG-3′ and reverse primer: 5′-GAAGGACAGGAATGGGAACA-3′: *β*-actin: forward primer: 5′-GGCTGTATTCCCCTCCATCG-3′ and reverse primer: 5′-CCAGTTGGTAACAATGCCATGT-3′.

### 2.8. Effects of Ephedra Aconite* Asarum* Decoction on the Expression of IL-12, IFN-*γ*, IL-6, IL-1*β*, IL-4, and IL-13 in LPS-Induced DCs

DCs in culture were plated into 24-well plate at 1 × 10^6^ cells/ml, 500 *μ*l per well, set 3 experimental groups including group C, group L, and group M. Groups L as the model control group and M were induced with LPS (1 *μ*g/ml, Sigma), and group M was also treated with Ephedra Aconite* Asarum* Decoction (2 mg/ml). Cell culture supernatant from groups C, L, and M was harvested after 24 hours and the levels of IL-12 IFN-*γ*, IL-6, IL-1*β*, IL-4, and IL-13 in the supernatant were detected on an AimPlex flow cytometer.

### 2.9. Statistical Analysis

Statistical analysis was performed with SPSS 20.0 statistical software. Experimental data were expressed as mean ± SD. One-way ANOVA was used for comparison among multiple groups, and multiple intergroup comparisons were performed using *q* test. *p* < 0.05 was set as the criterion for statistically significant difference.

## 3. Results

### 3.1. Culture and Flow Identification of IMDCs

**Figure 1:**
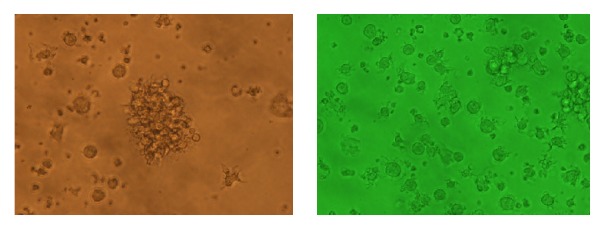
Bone marrow-derived IMDCs (20x eyepiece).

**Figure 2:**
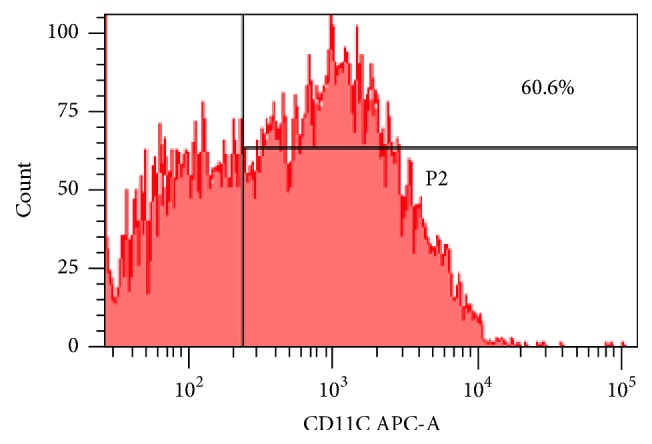
IL-4 and GM-CSF-induced bone marrow monocytes, the single peak P2 is CD11c^+^ DCs, so the purity of IMDCs was determined to be ~60%.

The induction of IL-4 and GM-CSF* in vitro* can invert bone marrow monocytes into IMDCs rapidly and efficiently (Figure 1). The IMDCs induced for 7 days have high purity (above 60%, Figure 2) and grow in multiple clusters with obvious processes. The yield is about 1.5 × 10^7^ IMDCs per mouse, which is sufficient for subsequent experiments.

### 3.2. CCK-8 Test to Determine the Toxicity of Ephedra Aconite* Asarum* Decoction Water Extract on DCs at 24 Hours after Treatment

**Figure 3:**
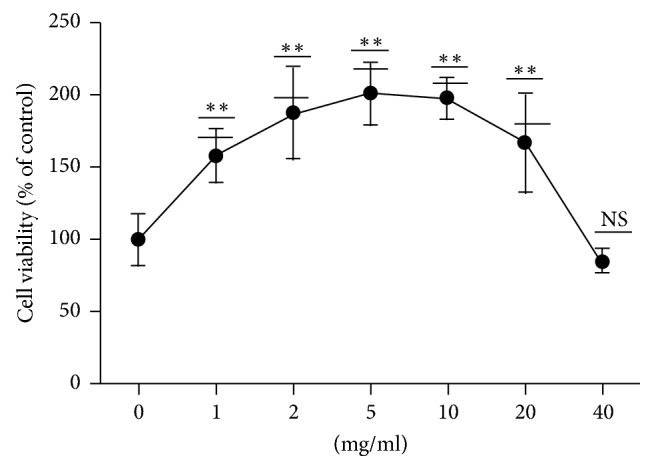
The effect of CCK-8 on DCs, ^*∗∗*^*p* < 0.01 as compared to the blank control (0 mg/ml), NS: no significant difference.

CCK-8 detection of the toxicity of Ephedra Aconite* Asarum* Decoction on DCs showed relatively low toxicity on DCs (Figure 3). The effects on cell viability were different within varying dose ranges. At a concentration of 5 mg/ml, CCK-8 detected the highest cell viability. When the drug concentration reached 10 mg/ml, although the cell viability was still higher than that of the normal group, it was lower than the peak value, so an inhibitory effect of the drug was shown. When the drug concentration reached 40 mg/ml, cell viability was not different from the normal group; that is, it decreased significantly from the peak, so drug toxicity was significant at high dose.

### 3.3. Effects of Ephedra Aconite* Asarum* Decoction on LPS-Induced DCs Maturation

To find out whether Ephedra Aconite* Asarum* Decoction could change the expression of CD80, MHCII, and CD83 on DCs after LPS stimulation, we analyzed the effect of the effect of this medicine DCs stimulated by LPS after 24 hours.

**Figure 4:**
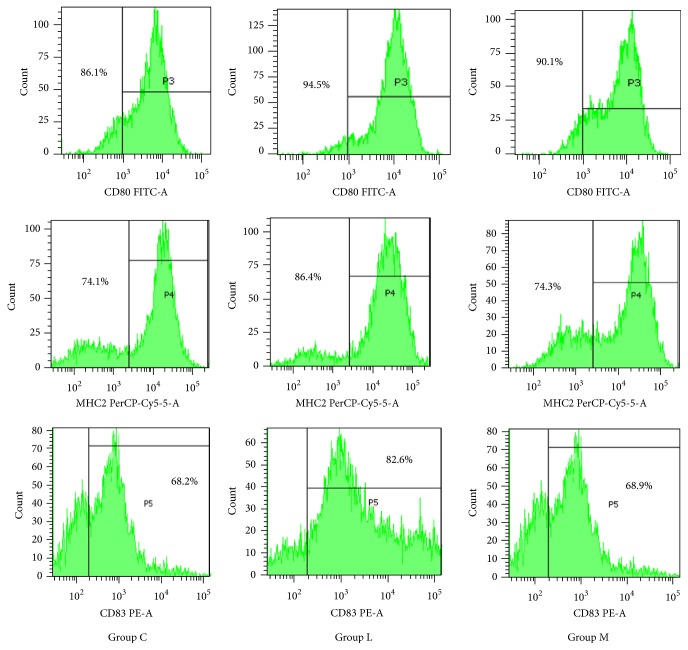
P3, P4, and P5 represent the expression of CD80, MHCII, and CD83 on DCs from group C, group L, and group M.

As shown in Figure 4, single peak represents the expression of CD80, MHCII, and CD83 in LPS-Induced DCs. The percentages of expression in group C were 86.1% of CD80, 74.1% of MHCII, and 68.2% of CD83. The percentages of expression in the LPS intervention group L were 94.5% of CD80, 86.4% of MHCII, and 82.6% of CD83. The percentages of expression in group M were 90.1% of CD80, 74.3% of MHCII, and 68.9% of CD83.

**Figure 5:**
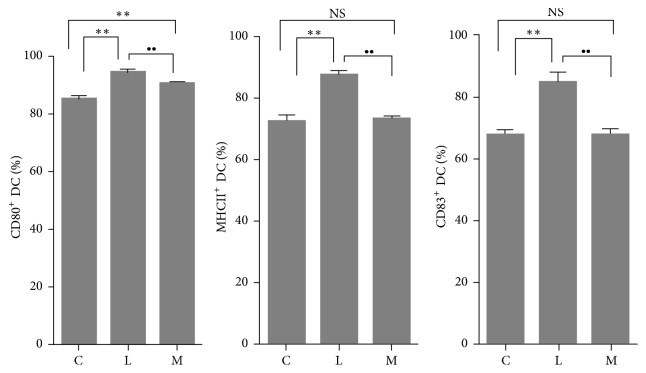
The percentage of CD80^+^ DC, MHCII^+^ DC, and CD83^+^ DC among total* in vitro* LPS-Induced DCs in groups C, L, and M, ^*∗∗*^*p* < 0.01 as compared with group C, ^∙∙^*p* < 0.01 as compared with group L, NS: no significant difference.

As shown in Figure 5, groups C, L, and M represent the ratios of CD80^+^ DCs, MHCII^+^ DCs, and CD83^+^ DCs to the total LPS-Induced DCs in group C. In group L, the percentages of CD80^+^ DCs, MHCII^+^ DCs, and CD83^+^ DCs were higher than those in group C (*p* < 0.01). In group M, the ratios of CD80^+^ DCs, MHCII^+^ DCs, and CD83^+^ DCs were all lower than those of group L (*p* < 0.01), while the proportion of CD80^+^ DCs was still higher than that in group C (*p* < 0.01). From these results, Ephedra Aconite* Asarum* Decoction lowed the expression of CD80, MHCII, and CD83 on DCs after LPS stimulation, inhibiting the maturation of LPS-DCs.

### 3.4. Effects of Ephedra Aconite* Asarum* Decoction on the Expression of IL-12, IFN-*γ*, IL-6, IL-1*β*, and IL-4 and in LPS-Induced DCs

**Figure 6:**
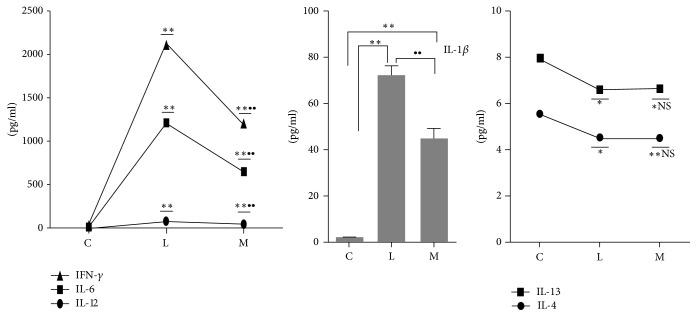
The expression status of IL-12, IFN-*γ*, IL-6, IL-1*β*, IL-4, and IL-13 in group C, group L, and group M, ^*∗*^*p* < 0.05 and ^*∗∗*^*p* < 0.01 as compared with group C; ^∙∙^*p* < 0.01 as compared with the model group (group L).

In order to detect the effect of Ephedra Aconite* Asarum* Decoction of anti-inflammatory after LPS stimulation, we analyzed Th1 cytokines including IL-12, IFN-*γ*, IL-6, and IL-1*β* and Th2 cytokines involving IL-4 and IL-13. All of them were contained in supernatant of DCs stimulated by LPS after 24 hours with or without Ephedra Aconite* Asarum* Decoction intervention (Figure 6). IL-12, IFN-*γ*, IL-6, and IL-1*β* cytokine production in the LPS intervention group (group L) was significantly higher than that in group C (*p* < 0.01), while Ephedra Aconite* Asarum* Decoction intervention significantly reduced the levels of cytokine (*p* < 0.01) as compared to group L, but the content still higher than that in group C (*p* < 0.01). The production of IL-4 and IL-13 in group M was lower than that in group C (*p* < 0.01), while it varied a little comparing to group L. It could be clearly seen that Ephedra Aconite* Asarum* Decoction inhibited the releasing of Th1 inflammatory factors in a short time. However, it possibly played an inactive role in the expressions of IL-4 and IL-13 after LPS intervention.

### 3.5. Effects of Ephedra Aconite* Asarum* Decoction on the Expression of STAT6 and pSTAT6 in LPS-Induced DCs

Since we observed that Ephedra Aconite* Asarum* Decoction can inhibit the maturation of LPS-DCs and inhibit the releasing of inflammatory cytokines of LPS-DCs, we further tested the upstream pathway of anti-inflammation effect of Ephedra Aconite* Asarum* Decoction.

**Figure 7:**
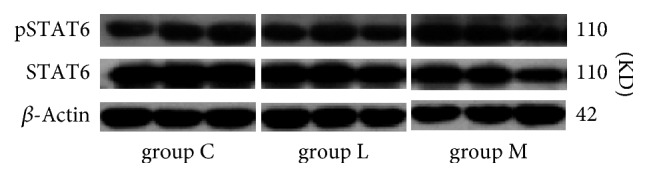
STAT6 and pSTAT6 protein bands in group C, group L, and group M, each group was made in triplicate. Each band was accompanied by a parallel control.

**Figure 8:**
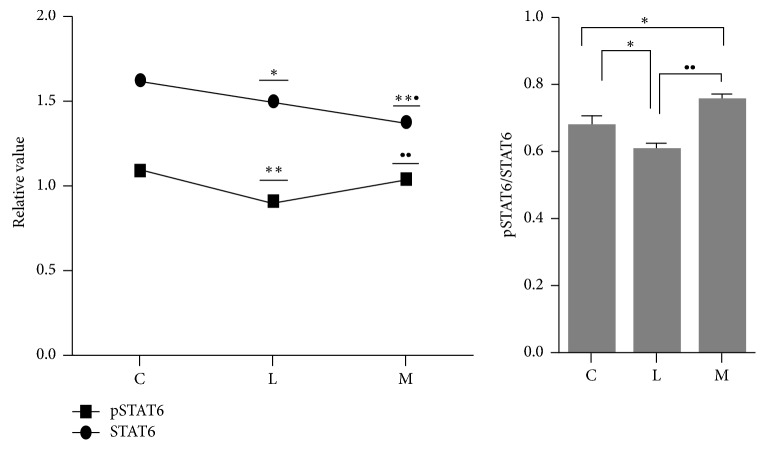
The expression of STAT6, pSTAT6, and pSTAT/STAT6 ratio in group C, group L, and group M, ^*∗*^*p* < 0.05, ^*∗∗*^*p* < 0.01 as compared with group C; ^∙^*p* < 0.05 and ^∙∙^*p* < 0.01 as compared with the model group (group L).

The results of WB bands were showed in Figure 7. WB analysis showed (Figure 8) that the expression of STAT6 in LPS intervention group (group L) was lower than that in group C (*p* < 0.05), while that in group M was decreased when compared to both group C and the LPS-induced group (*p* < 0.01; *p* < 0.05). The expression of pSTAT6 in the LPS group was lower than that in group C (*p* < 0.01) and that in group M was significantly higher than that of group L (*p* < 0.01), but not significantly different from that of group C. The ratio of pSTAT6/STAT6 in group L was lower than that in group C (*p* < 0.05), while that in group M was elevated as compared to both group C and the LPS-induced group (*p* < 0.05; *p* < 0.01). Ephedra Aconite* Asarum* Decoction can increase the level of expression of pSTAT6 associated with Th2, correcting the Th1 shift caused by LPS stimulation by regulating the DCs to induce Th2 differentiation.

### 3.6. Effects of Ephedra Aconite* Asarum* Decoction on GATA-3 and T-Bet mRNA Expression in LPS-Induced DCs

**Figure 9:**
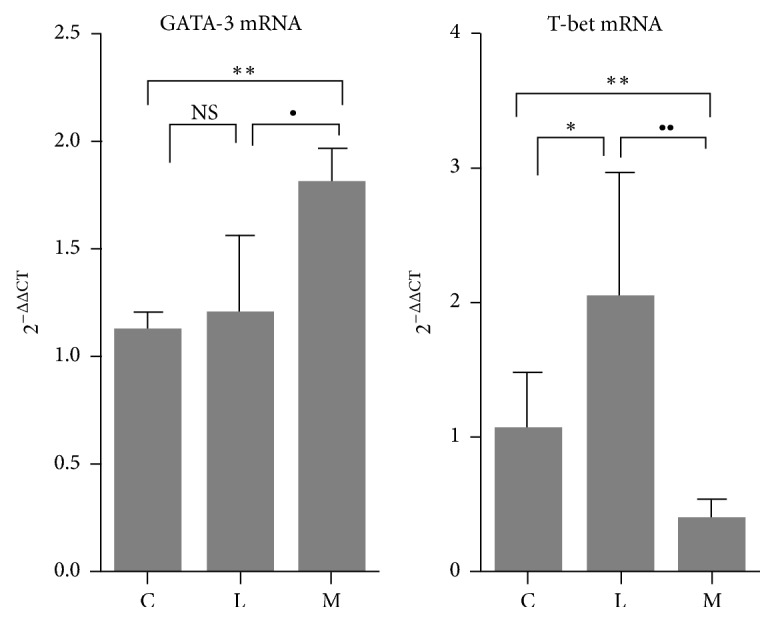
The expression of GATA-3 mRNA in group C, group L, and group M, ^*∗*^*p* < 0.05 and ^*∗∗*^*p* < 0.01 as compared with group C; ^∙^*p* < 0.05 and ^∙∙^*p* < 0.01 as compared with group L, NS: no significant difference.

We further analyzed GATA-3 mRNA, the upstream of STAT6, and Th1 directly related to T-bet mRNA (Figure 9). T-bet and GATA-3 are specific transcription factors for Th1 and Th2 cell differentiation, respectively. The expression of T-bet mRNA increased after LPS induction of DC as compared with group C (*p* < 0.05), while the expression of GATA-3 mRNA was not significantly different from group C. After intervention by Ephedra Aconite* Asarum* Decoction, the expression of T-bet mRNA was significantly decreased than that in the LPS group (group L) and group C (*p* < 0.01), while the expression of GATA-3 mRNA was increased as compared to group C and the LPS group (group L) (*p* < 0.01; *p* < 0.05). There was no significant difference in GATA-3 mRNA between the control and the LPS group. Ephedra Aconite* Asarum* Decoction can directly inhibit the transcription factor of T-bet and upregulate Th2 related transcription factors GATA-3, working together in regulation of Th1 inflammation induced by LPS stimulated DCs.

## 4. Discussion

### 4.1. Toxicity of Ephedra Aconite* Asarum* Decoction on DCs

The toxicity of Ephedra Aconite* Asarum* Decoction on DCs was detected by CCK-8 assay, which is relatively low as shown by our experimental results. When the concentration was within 20 mg/ml, viability of DCs increases with increased concentration of Ephedra Aconite* Asarum* Decoction. However, when the drug concentration is greater than 20 mg/ml, cell viability began to decline, and the promoting effect of Ephedra Aconite* Asarum* Decoction on cell viability gradually disappeared. When the Ephedra Aconite* Asarum* Decoction concentration is greater than 40 mg/ml, cell viability was significantly inhibited, that is, the drug had toxic side effect on DCs at that dose. In this study, we chose the concentration of 2 mg/ml of Ephedra Aconite* Asarum* Decoction for* in vitro *experiments on DCs.

### 4.2. Effects of Ephedra Aconite* Asarum* Decoction on the Maturation of LPS-Induced DCs

As shown by flow cytometry results, Ephedra Aconite* Asarum* Decoction had an inhibitory effect on LPS-Induced DCs maturation. After the activation of LPS, DCs quickly expressed high levels of CD83, CD80, MHCII, and other surface costimulators. The increased expression of MHCII enhanced the ability of DC to present exogenous antigens and promoted CD4^+^ T cell activity. CD80 and CD86 are synergistic costimulators that act as important second messengers to stimulate T cell function [34]. CD83 is an important membrane surface molecule of mature DCs, so the increase of CD83 expression not only activates DCs, but also provides costimulatory signals for both primary T cells and memory T cells [35]. Ephedra Aconite* Asarum* Decoction can significantly reduce the expression of CD83, CD80, and MHCII on LPS-Induced DCs, inhibit DC maturation, and reduce the ability of DCs to present antigens.

### 4.3. Effect of Ephedra Aconite* Asarum* Decoction on pSTAT6 in LPS-Induced DCs

STAT6 is a member of the STATs family and is present in the cytoplasm as an inactive monomer. STAT6 can be activated by IL-4 and IL-13 [14, 15]. Activation of STAT6 further induces secretion of Th2 cytokines, which means that STAT6 hyperphosphorylation provides an intimation of the Th2 type inflammation. Thus, as a key transcription factor for the induction of Th2-type allergic inflammation, STAT6 increases susceptibility to allergic inflammation and its role in many allergic diseases had grabbed the attention of many researchers. The activation of STAT6 in DCs is a key step in DCs-induced Th2 differentiation [36].

After the stimulation of LPS, pSTAT6 in DCs was significantly reduced, with the secretion of Th2 cytokines inhibited, thus creating a preferentially Th1 cytokine microenvironment. Ephedra Aconite* Asarum* Decoction can reverse pSTAT6 and thus has the potential to promote STAT6 activation. This modulates Th1 differentiation and regulates Th1 and Th2 balance.

### 4.4. Effects of Ephedra Aconite* Asarum* Decoction on GATA-3 mRNA and T-Bet mRNA Levels in LPS-Induced DCs

Recent studies have proved that GATA-3 mRNA and T-bet mRNA are also expressed on DCs [37, 38] to facilitate DCs-induced Th differentiation, although they are traditionally considered to be the most important transcription factors in T cells to determine Th1/Th2 differentiation [39, 40]. Once activated, T-bet promotes the secretion of Th1 cytokines and induces Th1 differentiation. As an upstream molecule of STAT6, its activation can trigger GATA-3 activation and thus induce the secretion of Th2 cytokines such as IL-5 and IL-13, playing a key role in differentiation of Th2 cells [41].

The expression of Tet mRNA in DCs was elevated after LPS stimulation, but the expression of GATA-3 mRNA was not significantly changed. After Ephedra Aconite* Asarum* Decoction intervention, T-bet mRNA significantly reduced while GATA-3 mRNA increased. These results suggest that Ephedra Aconite* Asarum* Decoction could reverse pSTAT6, promote GATA-3 mRNA expression, and regulate Th1 differentiation induced by LPS-Induced DCs. Overall, it might have the potential to restore Th1 and Th2 imbalance and further offer a new solution to Th1 type inflammation.

### 4.5. Effects of Ephedra Aconite* Asarum* Decoction on the Production of IL-12, IFN-*γ*, IL-6, and IL-1*β* in LPS-Induced DCs

IL-12, IFN-*γ*, IL-6, and IL-1*β* are very important proinflammatory and immunoregulatory factors secreted by the DCs during inflammatory response [42]. After the stimulation of LPS, DCs can promote the secretion of IL-12, IFN-*γ*, IL-6, and IL-1*β* through TLRs [43], p38 [44], and JAK-STAT [45] signaling pathways. In the process, IL-12 can further induce the secretion of IFN-*γ* by DCs and synergistically acts with IL-18 to induce T cell differentiation towards Th1 direction [46]. Furthermore, the increasing cytokines such as IL-12, IFN-*γ*, IL-6, and IL-1*β* will undoubtedly mediate or aggravate the inflammatory responses and induce T cells to differentiate towards the Th1 direction. With the intervention of Ephedra Aconite* Asarum* Decoction, secretion of the above cytokines was significantly reduced, and inflammation caused by these cytokines was inhibited. Meanwhile, cytokine-induced Th1 differentiation of T cell was reversed, so Ephedra Aconite* Asarum* Decoction showed a strong anti-inflammatory effect.

### 4.6. Effects of Ephedra Aconite* Asarum* Decoction on the Production of IL-4 and IL-13 in LPS-Induced DCs

IL-4 and IL-13, as the classic cytokines of Th2, were both related to GATA-3 mRNA and STAT6 [47], and they were supposed to have some obvious changes after the intervention of Ephedra Aconite* Asarum* Decoction on LPS-induced DCs. Nevertheless, little differences of the contents of IL-4 and IL-13 in group M could be distinguished comparing to the results of group L, whereas both contents of the cytokines were lower than those in group C. The results might mainly imply that Ephedra Aconite* Asarum* Decoction could influence other significant cytokines about Th2 which were secreting by LPS-Induced DCs, or there was not enough time for LPS-Induced DCs to secrete IL-4 and IL-13 after the intervention of Aconite* Asarum* Decoction. After all, just 24 hours were allowed for the interaction.

In summary, the results indicate that Ephedra Aconite* Asarum* Decoction can modulate LPS-induced Th1 skewing and show a rapid anti-inflammatory effect. The specific process is caused by blocking the maturation of LPS-induced IMDCs including downgrading T-bet mRNA expression, promoting pSTAT6, and reducing the overexpression of Th1 cytokines such like IL-1*β*, IL-12, IFN-*γ*, and IL-6 from DCs. At the same time, the data implies that Ephedra Aconite* Asarum* Decoction can upregulate the expression of GATA-3 mRNA in LPS-Induced DCs. However, as to the IL-4 and IL-13 relevant to GATA-3 mRNA, Ephedra Aconite* Asarum* Decoction has no apparent effect to upregulate them. Taken together, our findings may have a speculation that Ephedra Aconite* Asarum* Decoction can regulate immune responses and play an anti-inflammatory effect through regulating the GATA-3/STAT6 pathway in LPS-Induced DCs. Yet whether Ephedra Aconite* Asarum* Decoction can intervene in Th1 type inflammation through other pathways and whether there is an explicit effect of the decoction to benefit the secreting of Th2 cytokines are still worthy of further studies.
